# On-Device ECG and Vital-Sign Prediction of 24-Hour Mortality and Unplanned ICU Admission in Emergency Department Patients: A Retrospective Evaluation of MEDI-AI

**DOI:** 10.3390/diagnostics16193153

**Published:** 2026-09-28

**Authors:** Hyunjin Kyung, Dongbum Suh, Jin Hee Lee, Hyuksool Kwon

**Affiliations:** Department of Emergency Medicine, Seoul National University Bundang Hospital, Seongnam 13620, Republic of Korea; rud3565@naver.com (H.K.); dongbum@snubh.org (D.S.); gienee@snubh.org (J.H.L.)

**Keywords:** emergency department, electrocardiography, vital signs, mortality, intensive care, smartphone, clinical prediction, calibration

## Abstract

**Background/Objectives:** Smartphone-based analysis of electrocardiograms (ECGs) and vital signs may support risk assessment without an integrated electronic health record feed. We evaluated MEDI-AI for 24 h mortality and unplanned intensive care unit (ICU) admission, with secondary diagnostic and triage assessments. **Methods:** This single-center retrospective evaluation included 2000 encounters from 2025 after supplementary development on 3000 encounters from 2024, with patient-level separation. Stored probabilities were assessed for area under the receiver-operating-characteristic curve (AUROC), calibration, and threshold performance. Post hoc exploratory NEWS2 and paired comparisons were performed; accepted inputs were also analyzed separately. **Results:** There were 189 deaths and 378 ICU admissions. Fusion AUROCs were 0.9994 and 0.9982; NEWS2 AUROCs were 0.8801 and 0.8131. Fusion-minus-vital-only AUROC differences were −0.000039 (95% CI, −0.000450 to 0.000366) and 0.002114 (0.000632 to 0.003920). Fusion calibration slopes were 5.62 and 6.09. STEMI and ACS sensitivities were 90.9% (58.7–99.8%) and 92.3% (74.9–99.1%). Triage critical-class recall was 24.9%. Input rejection occurred in 48 encounters; median recorded latency was 1235 ms. These stored-score estimates do not measure end-to-end deployable performance. **Conclusions:** Near-ceiling stored-score discrimination did not establish reliable absolute risks or a clinically usable workflow. Incomplete predictor-timing and model-development records limit reproducibility and assessment of bias. The evaluated triage configuration is unsuitable for clinical deployment, and the diagnostic analyses do not validate rule-out use. Independent external and prospective validation are required.

## 1. Introduction

Short-term mortality and unplanned intensive care unit (ICU) admission are important outcomes in emergency department (ED) risk assessment. Physiological early-warning scores offer a structured approach to deterioration detection; the National Early Warning Score 2 (NEWS2) is an established example. ED comparisons of early-warning scores and machine-learning triage models illustrate the importance of evaluating discrimination, calibration, and the consequences of a selected operating threshold in the intended population [1,2,3]. These studies provide context rather than a head-to-head benchmark for the present cohort.

Recent NEWS2 evaluations have considered hospitalization, critical-care admission, and in-hospital mortality; their differing endpoints and settings favor direct, endpoint-matched comparison rather than cross-study rankings [4]. A methodological review of early-warning-score studies found that discrimination was reported much more often than calibration, reinforcing the need to evaluate both [5].

A photograph-based workflow offers a potential alternative when structured electrocardiogram (ECG) waveforms or vital-sign feeds are not directly accessible. Earlier paper-ECG digitization work established methods for converting image representations into analyzable signals [6]. More recent studies have evaluated scanned and smartphone-photographed ECGs: Wong et al. used smartphone capture with server-side processing, whereas Stenhede et al. reported an open-source digitization pipeline with acquisition-dependent reconstruction quality [7,8]. Sau et al. compared direct image-based mortality models with models using digitized waveforms, highlighting the importance of matching the input lead format [9]. Signal reconstruction, myocardial-infarction classification [10], and prognostic prediction remain separate tasks; success at one stage does not validate the complete clinical workflow.

Artificial intelligence (AI)-enhanced ECG is increasingly investigated as a digital biomarker platform spanning diagnosis, prognosis, and multimodal assessment, while validation and implementation gaps remain [11]. Prior studies have examined left ventricular systolic dysfunction [12], 1-year mortality [13], and risk estimation across international cohorts [14]. ED studies have also investigated 30-day mortality from standard 12-lead ECGs [15] and chest-pain stratification combining ECG, demographic, and cardiac-biomarker information [16]. These studies motivate investigation of ECG-derived information, but their populations, acquisition formats, and prediction horizons differ from a monitor-photograph-based 24 h ED task.

Recent emergency-care studies provide more direct multimodal precedents. Alcaraz et al. combined ECG waveforms with demographics, vital signs, laboratory measurements, and other clinical variables for deterioration and critical-outcome prediction [17]. Altintepe and Ozyoruk evaluated serial ECG waveforms with sequential vital signs and clinical features for hospital-admission prediction, an adjacent but different endpoint [18]. Image-based models have also undergone external evaluation for ED cardiac arrest and, separately, for short-term mortality across a large U.S. healthcare system [19,20]. Their acquisition formats, case selection, and outcomes differ from the present task, so their performance estimates are not cross-study benchmarks for MEDI-AI.

Triage urgency is a separate target from mortality risk. Evidence from established emergency triage systems shows heterogeneous performance, and NEWS2 prediction of time-critical treatment needs is not equivalent to mortality prediction [21,22]. Therefore, the prognostic, diagnostic, and triage components require separate evaluation.

MEDI-AI v1.0.4 is described as an iPhone-based system (Apple Inc., Cupertino, CA, USA) combining ECG digitization, vital-sign optical character recognition (OCR), prognostic models, acute coronary syndrome (ACS)/ST-elevation myocardial infarction (STEMI) screening, and triage classification. Its intended on-device implementation uses Core ML [23]. Framework support for local inference is an implementation feature, not evidence that privacy, clinical safety, or outcome benefit has been established for this application.

Actual local inference has been reported in other ECG applications. Wu et al. deployed an atrial-fibrillation-related model on an ECG device using images derived from digital waveforms [24]. Kartali et al. implemented local ECG processing and inference in an Android research platform [25]. These are technical on-device precedents, not validation of photographed-ECG and vital-sign prediction of ED deterioration.

We evaluated the recorded outputs of MEDI-AI in a temporally separated, single-center ED cohort. The primary objectives were prediction of 24 h mortality and unplanned ICU admission. Secondary objectives were ACS/STEMI screening, triage classification, input-quality rejection, and component/runtime performance. Reporting was organized using the TRIPOD (Transparent Reporting of a multivariable prediction model for Individual Prognosis Or Diagnosis) + AI update for prediction-model evaluation and STARD-AI (Standards for Reporting of Diagnostic Accuracy, extended for artificial intelligence) for the diagnostic component [26,27].

## 2. Materials and Methods

### 2.1. Study Design, Sampling Frame, and Encounter Eligibility

This retrospective study was conducted at Seoul National University Bundang Hospital, a tertiary academic center in the Republic of Korea. Supplementary model development used 3000 randomly sampled ED encounters from 2024. Evaluation used a separate random sample of 2000 encounters from a sampling frame of 45,078 adult ED visits in 2025. No additional eligibility filters were reported after this adult ED sampling frame was defined; pediatric visits were not eligible. Patient-level de-duplication across development and evaluation was applied. The evaluation cohort was not used for primary model fitting or threshold selection.

The available records identified the calendar years but not the exact within-year start and end dates or the sampling log. Observed evaluation ages ranged from 18 to 95 years; this observed range does not establish the original numerical age rule used to construct the adult sampling frame. The number of unique patients and the presence or frequency of repeated encounters within 2025 could not be recovered. The analysis unit was therefore the encounter. Cross-year de-duplication does not establish within-year independence; encounter-level confidence intervals may be too narrow if several encounters came from the same patient, and patient-clustered resampling was not possible.

Transfer and readmission status were not separately available, so their frequency and any original handling rule cannot be reported. Exclusion of baseline ICU status refers to the unplanned-ICU endpoint definition (Section 2.3), not a documented count of encounters removed from the entire cohort. No new eligibility exclusions, patient de-duplication, or relabeling were imposed during this reanalysis. Although the delivered numerical fields were complete, they do not establish the completeness of the original ECG or vital-sign acquisitions or identify records absent before extract construction. Supplementary Table S11 distinguishes these information gaps from the reported sampling and analysis rules.

Figure 1 separates the sampling frame, the random sample, quality status, and analysis populations. The 43,078 visits not sampled are the arithmetic complement of the 2000-encounter sample, not a count of clinical or technical exclusions. No delivered encounter was removed from the primary stored-score analysis. The 48 quality-rejected encounters were retained in that analysis, while accepted-input sensitivity analyses used 1952 encounters. Sample size was determined by the available evaluation cohort; no prospective power calculation was undertaken. Ethics and consent information is presented in the back matter.

### 2.2. Index System, Input Interface, and Prespecified Outputs

The described pipeline applies Apple Vision OCR to vital-sign monitor images, digitizes the ECG tracing, and generates ECG-only, vital-only, and fusion probabilities for each prognostic endpoint. The medi-ai-acs component produces STEMI and ACS-any scores. A separate three-class triage output (Normal, Urgent, Critical) is followed by a NEWS2-based fallback. The evaluated release is v1.0.4, developed by the Department of Emergency Medicine, Seoul National University Bundang Hospital. Positive classifications used probability ≥0.10 for mortality, ≥0.15 for ICU admission, ≥0.40 for STEMI, and ≥0.35 for ACS-any; these thresholds were reported as prespecified.

The numerical thresholds were used unchanged in this reanalysis. Their prespecification and the absence of threshold selection on the 2025 cohort remain source-author-reported study facts. A dated selection or freeze log, the selection criterion, and the exact development subset used to choose each threshold were not recoverable; these details were not inferred from the threshold values or retrospectively optimized.

The present analysis evaluated recorded component and fusion probabilities; it did not retrain the prognostic, diagnostic, or triage models. Available clinical descriptors included age, sex, heart rate, blood pressure, respiratory rate, oxygen saturation, temperature, Glasgow Coma Scale, and a recorded NEWS2 score. These analysis fields are not a verified list of the predictors actually entering each model. The evaluated release’s complete predictor definitions, component architectures, external pretraining sources, preprocessing and missing-input rules, hyperparameter-selection procedure, and fitted fusion rule could not be reconstructed from the available records. A recoverability inventory is provided in Supplementary Table S10. Same-cohort logistic recalibration was a separate exploratory analysis, not redevelopment or independent validation of the primary models.

The ECG inputs used for evaluation were photographs of ECG tracings displayed on actual monitors. The extract contained encounter-level scores and component-performance fields, not a complete image-acquisition log. The number of ECG photographs per encounter, the role or training of the person acquiring them, and whether repeated capture was permitted were not recoverable. One encounter must therefore not be interpreted as one image or one capture attempt. These unavailable parameters are itemized in Supplementary Table S12 rather than reconstructed from the representative interface.

The specified outcome horizon was 24 h after measurement. However, the available extract did not identify the timestamp operationalizing that measurement or link ECG and vital-sign measurements, image capture, processing, deterioration, treatment, and the decision for ICU transfer. The intended horizon must therefore be distinguished from a verified prediction-time anchor. The analysis cannot establish that every predictor was available before deterioration or the ICU-transfer decision; recognition of established critical illness cannot be separated from prediction of future deterioration. The smartphone model, operating-system build, and ECG lead configuration were unavailable. They were not inferred from the iPhone-based implementation description, interface guidance, or recorded latency. Runtime estimates apply only to the recorded, incompletely documented test configuration.

Figure 2 illustrates the ECG-image input and vital-sign entry controls using a representative web-rendered interface. The authors confirmed that the web layout corresponds visually to the application interface. The figure documents the input controls, rather than an archived screen of the evaluated release or a test of native on-device execution. No clinical data were entered, and no model inference was initiated to prepare the figure.

### 2.3. Reference Standards

Mortality was defined as death within 24 h of measurement, ascertained through time-stamped electronic medical record survival tracking. The ICU endpoint was unplanned ICU transfer within 24 h; baseline ICU status was excluded from the endpoint definition. ACS/STEMI reference diagnoses were established by a blinded dual-cardiologist consensus process using serial high-sensitivity troponin, coronary angiographic information, and the final discharge diagnosis. Triage was assessed by a blinded emergency-physician panel using Emergency Severity Index (ESI) version 4 [28]. Reference assessors were reported as blinded to model output.

The analysis used the recorded reference labels without re-adjudication or relabeling. The exact conversion of ESI v4 levels into Normal, Urgent, and Critical categories was not recoverable. No conventional or newly proposed ESI mapping was substituted for the recorded classes. The records also did not specify the handling of diagnostic disagreements or indeterminate cases. These gaps limit reproducibility of the secondary reference-standard comparisons; the confusion matrices describe the supplied reference categories only.

### 2.4. Analysis Populations and Missing or Rejected Inputs

The primary numerical analysis used stored probabilities and reference labels for all 2000 encounters. All required numerical fields were populated, and no values were imputed. A quality gate flagged 48 encounters as rejected despite their rows containing probabilities. All-encounter estimates are conditional retrospective stored-score benchmarks that may be optimistic for clinical use, not measurements of end-to-end sensitivity, workflow efficiency, or deployable output availability. Separate analyses of the 1952 accepted encounters and the rejected subset were retained. Acceptance alone does not establish successful bedside presentation or clinician use; rejected-subset estimates are descriptive because of their small size.

Image quality was represented in the extract by a binary acceptance/rejection flag. The underlying quality-assessment procedure, scoring rules, cutoffs, and whether a person or automated component assigned the flag were not recoverable. The 48 flagged encounters could not be separated reliably into ECG-image failure, vital-sign OCR failure, or other causes. Records did not establish whether failed captures were repeated, whether rejected-input scores were suppressed or displayed, or whether a fallback or clinician override was used. These scores remain only in explicitly labeled retrospective analyses and are not counted as valid clinical outputs. Source records absent before extract construction and failed capture attempts were not enumerated; their counts must not be assumed to be zero (Supplementary Table S12).

### 2.5. Statistical Analysis

Continuous variables are summarized as medians and interquartile ranges; categorical variables retain their denominators. Discrimination was measured by the area under the receiver-operating-characteristic curve (AUROC). Precision–recall performance was summarized by non-interpolated average precision (AP), not trapezoidal integration. The no-skill prevalence references for the all-encounter analyses were 0.0945 for mortality, 0.1890 for ICU admission, 0.0055 for STEMI, and 0.0130 for ACS-any; AP values must be interpreted relative to the corresponding outcome prevalence. Sensitivity, specificity, positive predictive value (PPV), negative predictive value (NPV), and false-negative rate were calculated at the fixed thresholds. Two-sided 95% confidence intervals (CIs) for proportions used the exact Clopper–Pearson method [29,30].

Uncertainty in the original model estimates was assessed with an ordinary encounter-level percentile bootstrap using 800 resamples and the original reproducible seed offsets from 20260910, without model refitting [31]. Those original estimates were retained. As a sensitivity analysis, bootstrap intervals were also computed with resampling stratified by outcome status (Supplementary Table S7); these intervals hold the observed numbers of events and non-events fixed. For post hoc NEWS2 and paired comparisons, 800 ordinary bootstrap samples used seed 20260923, reset for each analysis population. Within each replicate, identical encounter indices were used for every compared score and both outcomes; 2.5th and 97.5th percentiles used linear interpolation. An endpoint replicate containing only one outcome class would be skipped for every compared score; none was skipped. All intervals assume independent encounters rather than patient clusters. Paired comparisons are exploratory, unadjusted for multiplicity, and conditional on fixed predictions; they are not confirmatory superiority or clinical-benefit tests.

The supplied NEWS2 score was compared directly with recorded model scores for each endpoint, in all 2000 encounters and separately in the 1952 accepted-input encounters. NEWS2 thresholds of ≥5 and ≥7 were selected post hoc as conventional aggregate-score escalation cutoffs [1], not as previously prespecified MEDI-AI tests or independently validated action thresholds for this cohort. NEWS2 was neither reconstructed from the available vital signs nor converted into a predicted probability. The source lacked oxygen-scale selection, oxygen-treatment, and new-confusion/ACVPU fields, so the correctness of the supplied score construction could not be independently established; Glasgow Coma Scale was not substituted for these missing components. Paired AUROC differences were defined as fusion minus comparator.

Calibration was assessed with raw Brier scores, observed-to-expected event ratios, and equal-sized risk-decile groups formed by stable score rank, with ties allowed to split across adjacent groups. Exact binomial CIs describe the observed proportion in each decile. Logistic calibration estimated a joint intercept and slope from logit(P[Y = 1]) = a + b·logit(p). Calibration-in-the-large was estimated separately by an intercept-only model with logit(p) as an offset; it is not the joint intercept [32]. A slope above one indicates insufficient spread on the logit scale, while calibration-in-the-large describes overall over- or underprediction. Unpenalized logistic recalibration was fitted and evaluated on this same cohort only as an exploratory apparent-performance analysis, not as independent validation. Assessment across the risk range is important because mean calibration and calibration slope alone do not establish agreement at every probability [33].

Decision curves require a defined clinical action and a defensible threshold-based trade-off between true-positive and false-positive decisions [34]. Independently validated action thresholds and externally validated recalibrated mappings were not available for this workflow. Accordingly, decision curves and numerical net-benefit claims are not reported. Triage metrics included confusion matrices, macro-F1, Cohen’s kappa, and the change after fallback. OCR errors used paired recorded reference and OCR fields, both over all encounters and conditional on mismatch. Runtime quantiles used linear interpolation. Analyses were performed in Python (Python Software Foundation, Wilmington, DE, USA; https://www.python.org, accessed on 26 September 2026). 

Quantitative tables and performance figures were generated from a single aggregate result file to maintain numerical consistency; the representative input-interface image was captured separately as described in Figure 2. Estimates reported in the manuscript and supplementary tables were calculated using the stated statistical methods. The source summaries and the recomputed estimates were retained separately in the analysis records for traceability.

The generative-AI tool identified in the Acknowledgments assisted with revision text, analysis code, and aggregate-data visualizations. Statistical results were obtained by executing analysis code on the study extract and checked using separate computational implementations; generative AI was not used to synthesize patient-level observations or model predictions.

## 3. Results

### 3.1. Cohort and Input-Quality Accounting

The evaluation cohort included 2000 encounters, with mean age 62.1 years (range 18–95) and male sex recorded in 1083 encounters (54.1%). Table 1 reports baseline characteristics. Mortality occurred in 189/2000 encounters (9.45%), and unplanned ICU admission in 378/2000 (18.90%). There were 11 STEMI and 26 ACS-any reference diagnoses; these diagnostic categories are not summed as independent event groups.

The quality-acceptance fraction was 1952/2000, or 97.6% (95% CI, 96.8–98.2%). The rejected subset included three deaths and ten ICU admissions. Retained probabilities do not establish that a usable output was displayed, and these event counts must not be interpreted as successful detection by the full workflow. Accepted-input performance is reported separately in Supplementary Table S3; the dataset did not support a cause-specific rejection distribution.

### 3.2. Prognostic Discrimination and Operating Points

Fusion AUROC was 0.9994 (0.9989–0.9999) for mortality and 0.9982 (0.9972–0.9990) for ICU admission. Corresponding AP values were 0.9953 and 0.9925. Table 2 presents model and NEWS2 results; Figure 3 retains the three model input configurations. Mortality AUROC was numerically similar for vital-only (0.9995) and fusion (0.9994) scores. For ICU admission, the corresponding values were 0.9961 and 0.9982. These are descriptive comparisons, not evidence of incremental clinical benefit from ECG fusion.

In the post hoc direct comparison, NEWS2 AUROC was 0.8801 (0.8562–0.9019) for mortality and 0.8131 (0.7916–0.8345) for ICU admission; AP was 0.4387 (0.3703–0.5045) and 0.4701 (0.4271–0.5232), respectively. Fusion-minus-NEWS2 AUROC differences were 0.119332 (0.097721–0.143263) and 0.185134 (0.164124–0.205972). At NEWS2 ≥ 5, sensitivity/specificity were 92.1%/64.2% for mortality and 81.2%/68.2% for ICU admission; at NEWS2 ≥ 7, they were 87.8%/74.2% and 71.2%/77.6%. Exact intervals and confusion counts are reported in Supplementary Table S8.

Fusion-minus-vital-only AUROC differences were −0.000039 (−0.000450 to 0.000366) for mortality and 0.002114 (0.000632 to 0.003920) for ICU admission. The mortality interval includes zero; the positive ICU difference is small in absolute AUROC terms. Accepted-input sensitivity analyses are reported in Supplementary Table S9. These exploratory stored-score comparisons do not establish incremental clinical usefulness, validate predictor timing, or demonstrate that ECG acquisition is necessary.

At the prespecified thresholds, fusion sensitivity was 100.0% (95% CI, 98.1–100.0%) for mortality and 100.0% (95% CI, 99.0–100.0%) for ICU admission. No observed events were missed in the stored-score analysis. However, 501 mortality non-events and 608 ICU non-events tested positive, yielding PPVs of 27.4% and 38.3%, respectively. The ECG-only outputs missed twelve deaths and nine ICU admissions at the same outcome-specific thresholds. Complete operating-point estimates are in Table 3 and Supplementary Table S1.

### 3.3. Calibration and Exploratory Recalibration

Raw fusion probabilities were substantially miscalibrated (Table 4). Joint logistic calibration slopes were 5.62 for mortality and 6.09 for ICU admission, versus an ideal slope of 1. Separately estimated calibration-in-the-large intercepts were −0.602 and −0.558, respectively, and observed-to-expected ratios were 0.684 and 0.740. Under-dispersion on the logit scale therefore coexisted with overall overprediction; these raw outputs should not be communicated as calibrated individual risks or used directly as automated clinical triggers. Outcomes were highly concentrated: 186/189 deaths occurred in the highest mortality-risk decile, and 369/378 ICU events occurred in the two highest ICU-risk deciles, including 200/200 events in the highest decile. These observations describe the stored-score distribution; they do not by themselves establish absence or presence of leakage. Supplementary Table S2 provides all equal-rank deciles.

Exploratory same-cohort recalibration reduced fusion Brier scores from 0.0212 to 0.0043 for mortality and from 0.0440 to 0.0132 for ICU admission. Because the mapping was fitted and evaluated using the same outcome labels, this improvement is apparent performance, not evidence of a validated replacement probability scale. Figure 4 shows calibration only. Neither the original raw probabilities nor the same-cohort recalibrated mappings are validated for bedside absolute-risk communication or automated action.

### 3.4. Exploratory ACS/STEMI Assessment and Separate Triage Evaluation

The STEMI threshold detected 10/11 cases (sensitivity 90.9% (95% CI, 58.7–99.8%)), and the ACS-any threshold detected 24/26 cases (92.3% (95% CI, 74.9–99.1%)). Neither produced a false positive in the recorded sample (Table 5). Both scores had AUROC and AP of 1.0000, despite threshold false negatives: rank discrimination and threshold classification are different properties. Observed miss rates were 9.09% and 7.69%, respectively, but their exact upper 95% limits were 41.3% and 25.1%. These data therefore do not establish a population miss rate below 10%. STEMI and ACS-any misses must not be added without accounting for overlap.

Final triage accuracy was 43.2%, macro-F1 0.385, and Cohen’s kappa 0.100. Critical-class recall was only 24.9% (92/370). The fallback changed 16 outputs from Normal to Urgent: eight reference-Critical, four reference-Urgent, and four reference-Normal encounters. Critical recall did not improve. This degree of critical-class under-identification makes the evaluated triage configuration unsuitable for clinical deployment. It is a separate negative result and is not remedied by the prognostic models’ high AUROCs (Figure 5; Supplementary Table S4).

### 3.5. OCR and Recorded Runtime

Per-field OCR exact agreement ranged from 91.3% to 98.5%. Heart-rate mean absolute error (MAE) was 0.119 beats/min across all encounters and 2.67 beats/min among mismatches. Errors are reported in field-specific units; pooled numerical MAE across different vital-sign units would not be meaningful (Supplementary Table S5).

Median recorded application latency was 1235 ms, with 95th percentile 1602.1 ms and maximum 1942 ms. Runtime and memory summaries are provided in Supplementary Table S6. Separate source-reported component benchmarks, including ECG digitization, are retained in Supplementary Note S1 because their underlying paired component measurements are not represented in the encounter-level analysis. An offline-operation assertion is not treated as a network-egress audit.

## 4. Discussion

Our principal finding is the coexistence of near-ceiling discrimination, substantial miscalibration, and important limitations in the clinical interpretation of stored mortality and ICU-risk scores. A high AUROC describes ranking within this cohort; it does not establish accurate absolute risk, actionable advance warning, or clinical benefit. The moderate specificity and low PPV at the high-sensitivity thresholds imply a substantial potential alert burden. Uncertain predictor timing and incomplete development documentation are particularly consequential when discrimination is this high. These results should be treated as conditional retrospective benchmarks, not as evidence that the full smartphone workflow is safe or efficient.

The vital-only mortality model performed at least as well numerically as fusion, and the paired AUROC-difference interval includes zero. The observed ICU gain from fusion was small, even though its exploratory interval was above zero. Thus, ECG fusion did not demonstrate a mortality discrimination advantage, and its additional acquisition, digitization, and quality-control burden is not justified by an established clinical benefit in this study. Fusion ranked the stored endpoints better than the supplied NEWS2 score, but neither that comparison nor the broader AI-ECG literature establishes that the complex pipeline changes decisions or outcomes. A simpler physiological workflow remains an essential comparator in independent, timing-anchored validation [2,3].

The recent literature helps separate methodological progress from evidence of clinical usefulness. Multimodal ECG–clinical studies support testing complementary inputs, but hospital admission, cardiac arrest, and short-term mortality are not interchangeable targets [17,18,19]. External ECG-image evaluation can strengthen evidence about discrimination without establishing calibration or changed clinical outcomes; Lee et al. explicitly distinguished external discrimination validation from clinical implementation [20]. Our own paired comparisons, rather than performance values from those different cohorts, determine what can be said about the observed increment from ECG fusion.

The calibration analysis separates insufficient spread of predicted logits from overall overprediction. For both fusion endpoints, slopes above one coexisted with observed-to-expected ratios below one [32]. Raw model outputs should therefore not be presented to patients or clinicians as calibrated absolute risks, nor used unchanged to trigger automated decisions. A recalibration mapping would need to be fitted in suitable independent data and then tested in a separate validation sample before clinical use; simply fitting and assessing a mapping on the same cohort does not meet that requirement. Validated action thresholds and prospective assessment of changed decisions, false alerts, missed events, and harm are also needed. In their absence, decision curves and net-benefit claims are not reported, to avoid equating retrospective modeling with clinical utility. External-validation guidance distinguishes testing an existing model in new relevant data from refitting and assessing it on the same data [35].

The diagnostic analyses remain exploratory. Only 11 STEMI and 26 ACS-any events were available; the respective exact sensitivity intervals were 58.7–99.8% and 74.9–99.1%, and the upper confidence limits for the missed-case proportions were 41.3% and 25.1%. Thus, perfect ranking and high sample NPV do not establish a sufficiently low population miss rate. These data do not validate a stand-alone rule-out strategy, a safety-screen guarantee, or unsupervised disposition decisions. The triage module must be considered separately: poor critical-class recall persisted after fallback, and this configuration is unsuitable for clinical deployment regardless of prognostic discrimination. Subsequent diagnostic studies should plan sufficient disease-positive and disease-negative samples rather than rely on the total encounter count alone [36]. Prognostic external-validation sample sizes should likewise target the precision required for the intended setting [37].

The proposed smartphone workflow requires prospective end-to-end evaluation. A stored probability, even in an accepted-input encounter, does not establish a timely, correctly displayed, clinically usable result. Future evaluation should record sampling dates, unique patient identifiers with encounter linkage, the prespecified index time, each predictor’s measurement and availability times, photograph and processing times, deterioration and ICU-decision times, quality-rejection causes, repeat captures, overrides, no-output events, and clinical actions. Processing latency is not the total time from attempted capture to a usable result. On-device inference may reduce the need for server transmission, but privacy requires verification of the complete application’s data flow, storage, telemetry, and access controls; framework choice alone is insufficient. After independent offline validation, DECIDE-AI can guide early-stage live assessment of safety, clinician interaction, and workflow failure [38]. A subsequent randomized evaluation of clinical impact would require appropriate trial methods and reporting, including CONSORT-AI [39]. These are future stages, not evaluations completed in this study.

Acquisition-specific reporting is also essential. Recent digitization work found different reconstruction quality for scanned and smartphone-photographed ECGs, while image-based mortality modeling highlighted the relevance of the lead-display format [8,9]. Evidence of local execution on an ECG device or Android prototype does not establish the same execution, capture reliability, or clinical effectiveness for another application [24,25]. The missing acquisition log in this study therefore limits both technical reproducibility and interpretation of end-to-end workflow performance.

### Limitations

First, this was a retrospective, single-center evaluation. Temporal and patient-level separation between development and evaluation do not establish transportability or verify the temporal ordering of individual predictors and outcomes. The extreme concentration of outcomes in the highest score groups requires particular caution: the available records cannot exclude post-deterioration information or establish that prediction preceded the ICU-transfer decision. The findings may therefore partly reflect recognition of established critical illness rather than advance warning. This is a limitation of the available timing documentation, not proof that leakage occurred. Leakage and risk-of-bias assessment require examination of information availability and the complete preprocessing, development, and evaluation pathway, not cohort labels alone [40,41].

Second, the complete predictor set, trained model architecture and fusion rule, development settings, threshold-selection audit trail, exact sampling dates, and within-year patient linkage were not recoverable. Development/evaluation separation was applied as described in Section 2.1, but these additional gaps prevent complete independent reconstruction and a low-risk-of-bias conclusion. Encounter-level bootstrap intervals are conditional on fixed predictions and assume independence; they do not incorporate model-development uncertainty or unmeasured within-patient correlation. Same-cohort recalibration is apparent performance, and no external or prospective clinical validation was performed.

Third, unavailable cohort-construction and acquisition details limit assessment of selection and workflow-related bias. Transfer/readmission handling, the original numerical adult-eligibility rule, image counts, capture operators, repeat-capture permission, and quality-gate rules cannot be reconstructed. Complete stored numerical fields do not prove that every attempted clinical acquisition succeeded or that incomplete source records were absent. Explicit reporting of these gaps improves transparency but does not resolve them.

Finally, presenting diagnoses, major diagnostic categories, and comorbidities were unavailable for case-mix characterization. Identical age medians and interquartile ranges across outcome groups do not establish clinical homogeneity or age–outcome independence. High mortality and ICU-event prevalence and marked physiological differences limit generalization to other ED populations. Diagnostic events were sparse, ESI-to-category mapping and rejection causes were unavailable, and neither stored-score nor accepted-input analyses establish end-to-end clinical sensitivity.

## 5. Conclusions

In this retrospective temporally separated ED evaluation, recorded ECG–vital-sign and vital-only prognostic scores showed near-ceiling discrimination but substantial miscalibration and unresolved reproducibility and predictor-timing limitations. These results do not establish calibrated individual risks, advance warning, or clinical benefit. The diagnostic analyses are exploratory and do not validate rule-out safety; the evaluated triage configuration is unsuitable for clinical deployment. Independent external validation, appropriate recalibration, and prospective end-to-end evaluation are prerequisites to considering clinical use.

## Figures and Tables

**Figure 1:**
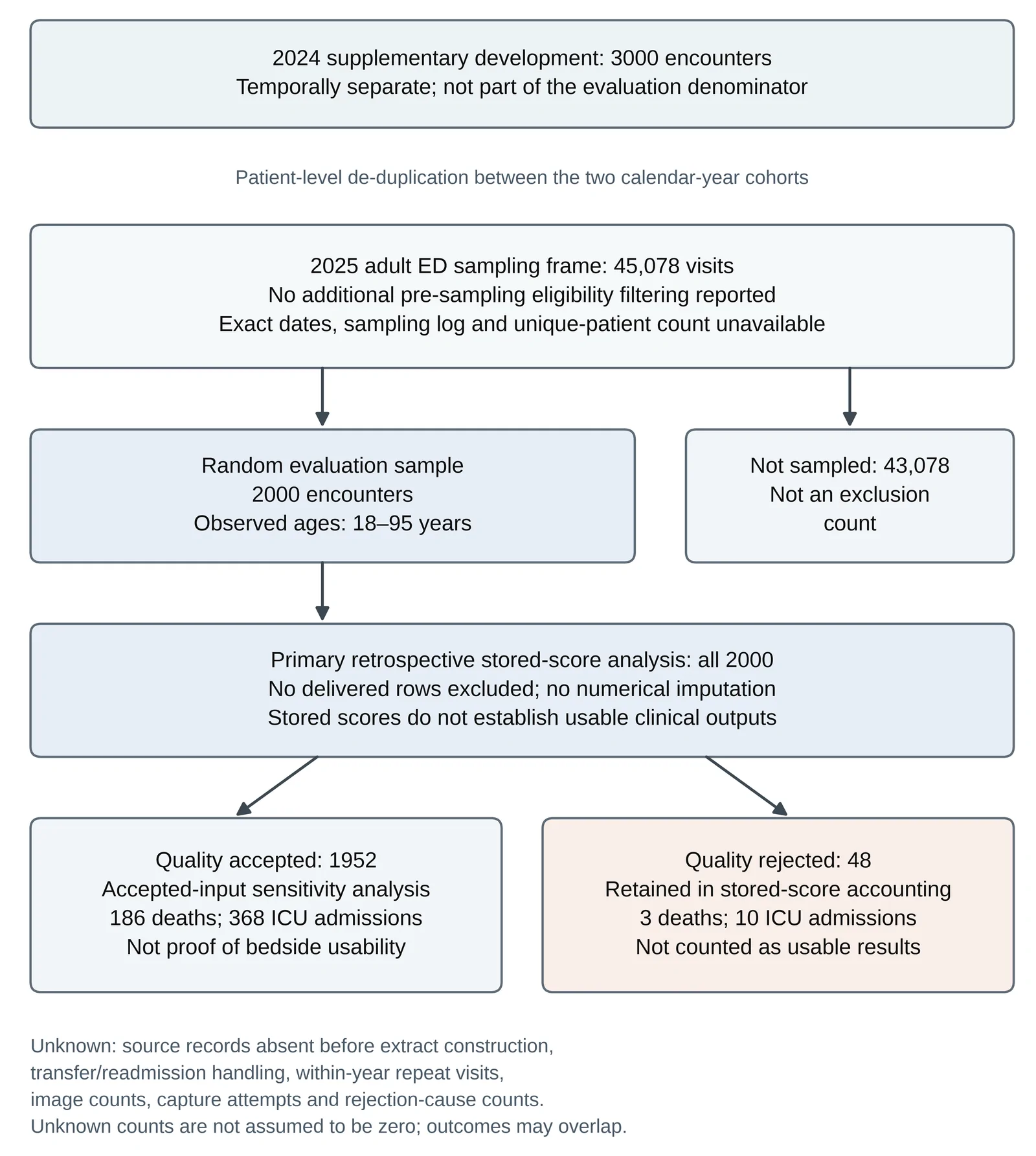
Participant and encounter accounting. The 2024 development cohort is separate from the 2025 evaluation denominator. Cross-year patient de-duplication does not establish the number of unique patients or repeated encounters within the evaluation year. “Not sampled” is distinct from “excluded”. All 2000 delivered encounters contribute to retrospective stored-score analyses, including 48 quality rejections; accepted-input sensitivity analyses use 1952 encounters. Unavailable upstream or acquisition counts are not assumed to be zero. Mortality and ICU outcomes may overlap; stored scores do not establish usable clinical outputs.

**Figure 2:**
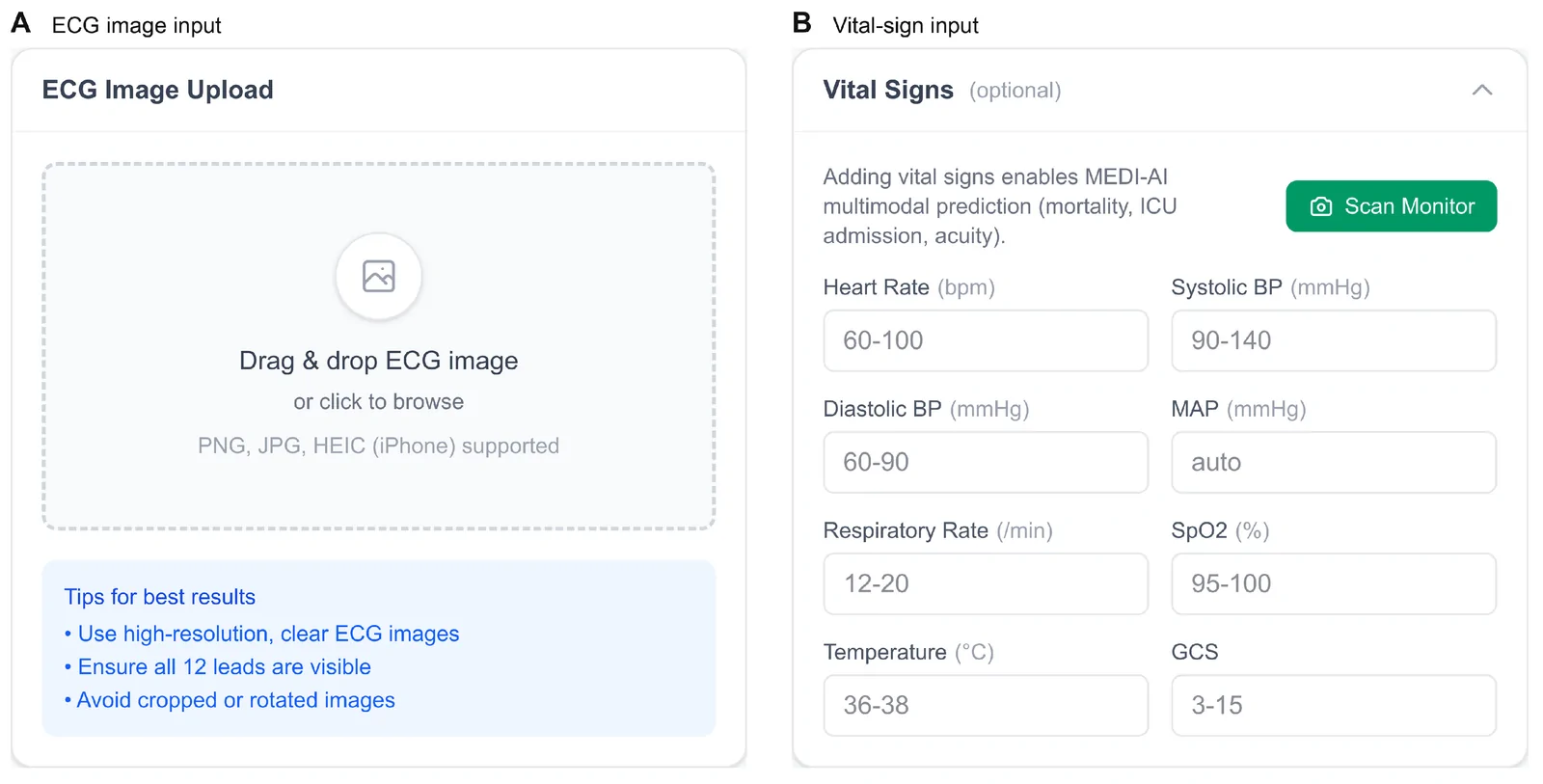
Representative MEDI-AI input interface. (**A**) ECG-image input panel. (**B**) Vital-sign entry panel, including the Scan Monitor control. Complete input cards were captured from https://ecg-webapp.vercel.app/ on 10 September 2026. Numeric ranges are empty-field placeholders, not participant measurements; no analysis output is shown. The generic 12-lead advice in panel A is interface guidance, not documentation of the study ECG lead configuration. BP, blood pressure; MAP, mean arterial pressure; SpO_2_, peripheral oxygen saturation; GCS, Glasgow Coma Scale.

**Figure 3:**
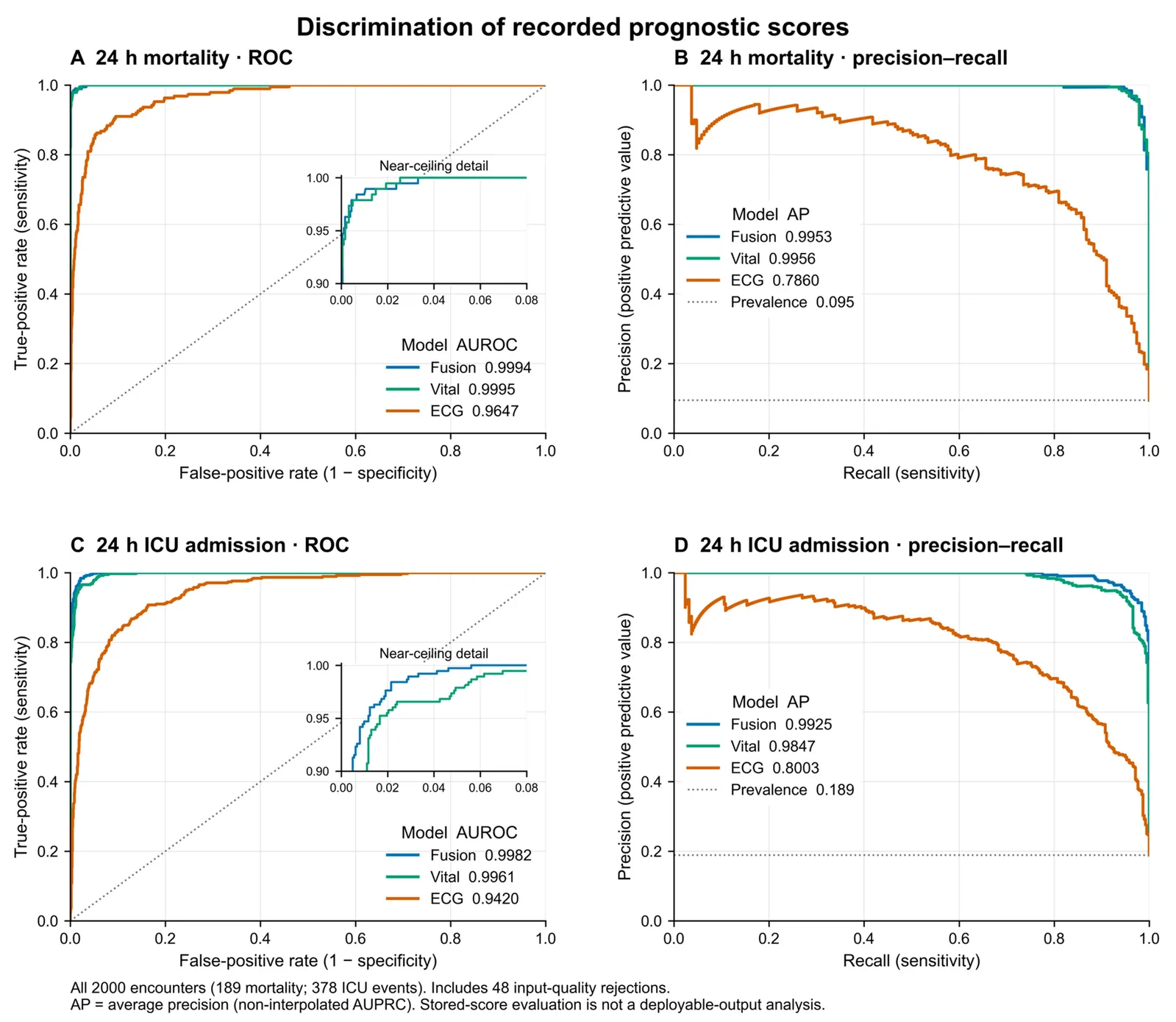
Discrimination of recorded prognostic probabilities. (**A**,**C**) ROC curves for 24 h mortality and ICU admission, with inset detail of the high-sensitivity region; the main axes span 0–1. (**B**,**D**) Precision–recall curves for the same outcomes. Legends report AUROC or non-interpolated AP. Dotted horizontal precision–recall references indicate outcome prevalence. Dotted diagonal lines in (**A**,**C**) indicate no discrimination (AUROC = 0.5). All 2000 encounters, including 48 flagged quality rejections, are represented.

**Figure 4:**
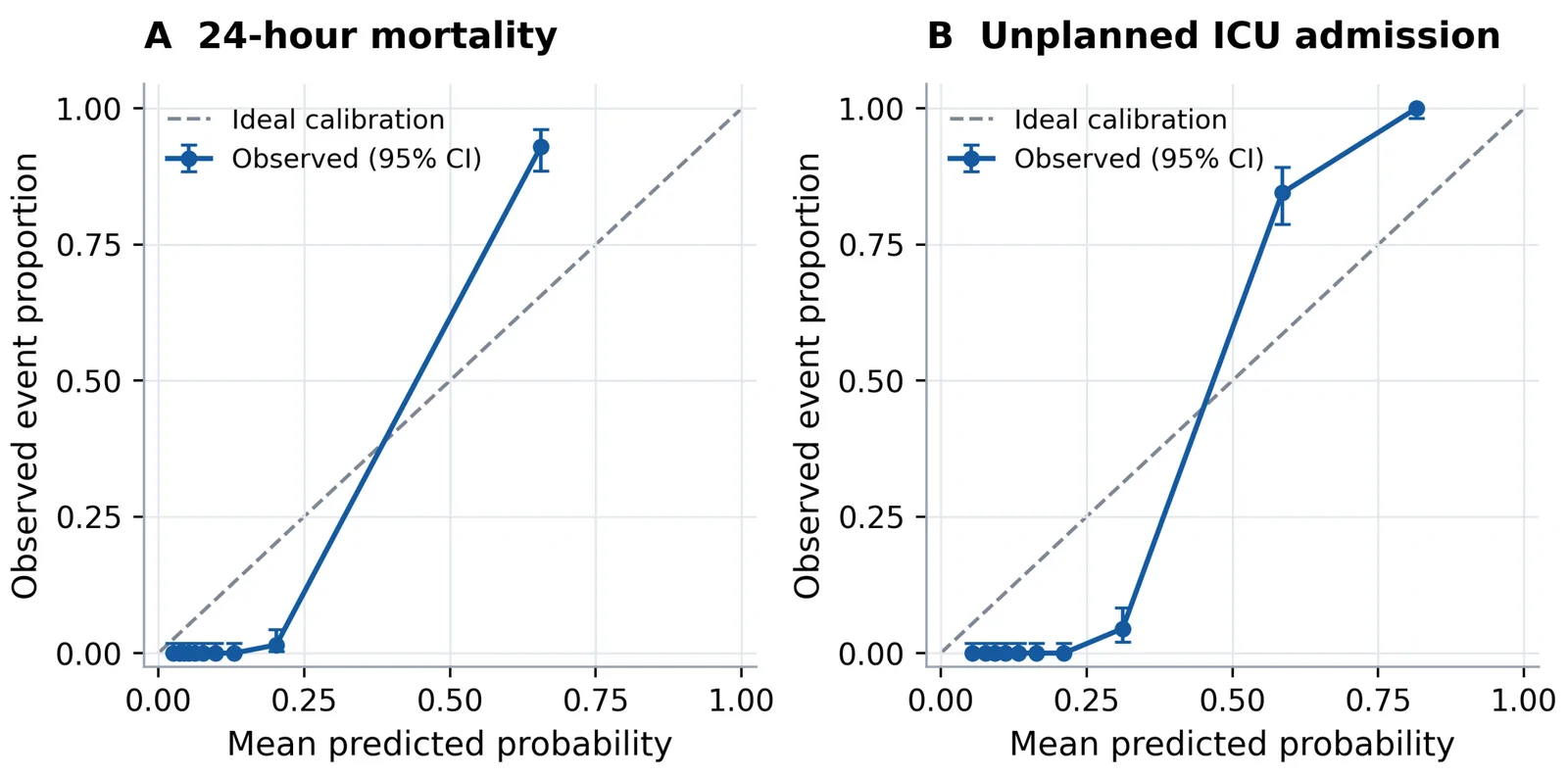
Calibration of recorded fusion probabilities. (**A**) 24 h mortality. (**B**) Unplanned ICU admission. Mean predicted probabilities and observed outcome proportions are shown in ten equal-rank groups of 200 encounters, with exact 95% binomial confidence intervals. Dashed diagonals indicate ideal calibration. All 2000 stored-score encounters, including quality rejections, are included. These are calibration descriptions, not validated clinical risk thresholds.

**Figure 5:**
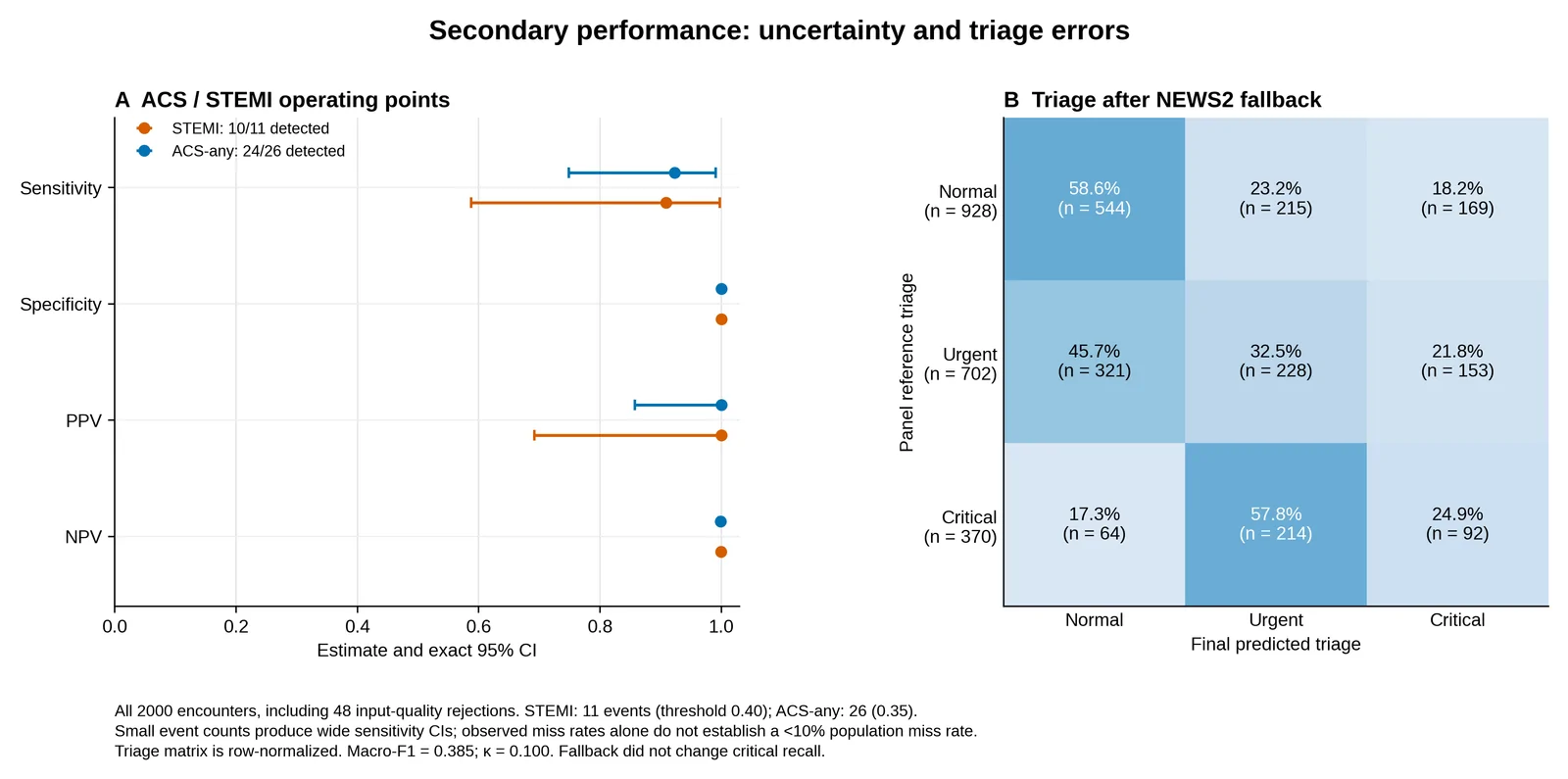
Secondary performance and uncertainty. (**A**) Exact 95% CIs for diagnostic sensitivity, specificity, PPV, and NPV; the small event denominators make sensitivity imprecise. (**B**) Final triage confusion matrix after fallback, with row-normalized percentages and encounter counts. Cell shading intensity is proportional to the row-normalized percentage. The fallback did not increase critical-class recall. Analyses include all 2000 stored-score encounters, including quality rejections.

**Table 1:** Baseline characteristics of the evaluation encounters.

Characteristic	All (*n* = 2000)	Death (*n* = 189)	No death (*n* = 1811)	ICU (*n* = 378)
Age, years	62 (53–71)	62 (53–71)	62 (53–71)	62 (53–71)
Male sex, *n* (%)	1083 (54.1)	101 (53.4)	982 (54.2)	201 (53.2)
Heart rate, /min	87 (76–99)	109 (99–120)	85 (75–96)	103 (93–113)
Systolic BP, mmHg	117 (105–129)	100 (88–114)	118 (107–129)	107 (95–117)
Mean arterial pressure, mmHg	86.7 (77.7–96.3)	74.7 (64.7–85.3)	87.7 (79.3–97.0)	79.3 (70.4–88.3)
Respiratory rate, /min	20 (17–23)	26 (23–29)	19 (16–22)	24 (21–27)
SpO_2_, %	96 (94–97)	92 (90–93)	96 (94–98)	93 (91–95)
Temperature, °C	36.8 (36.5–37.2)	37.3 (37.0–37.6)	36.7 (36.4–37.1)	37.2 (36.8–37.6)
Glasgow Coma Scale	15 (13–15)	11 (9–13)	15 (14–15)	13 (11–14)
NEWS2 score	3 (1–8)	11 (9–13)	3 (0–7)	9 (6–12)

Values are median (interquartile range), unless specified. ICU and mortality groups may overlap. Similar group-level age summaries do not establish statistical independence from outcome. BP, blood pressure; SpO_2_, peripheral oxygen saturation; NEWS2, National Early Warning Score 2.

**Table 2:** Discrimination of recorded prognostic scores and NEWS2.

Outcome/Input	AUROC (95% CI)	AP (95% CI)	Brier Score
Mortality/Fusion	0.9994 (0.9989–0.9999)	0.9953 (0.9909–0.9986)	0.0212
Mortality/Vital	0.9995 (0.9989–0.9999)	0.9956 (0.9909–0.9987)	0.0224
Mortality/ECG	0.9647 (0.9529–0.9740)	0.7860 (0.7224–0.8408)	0.0594
Mortality/NEWS2	0.8801 (0.8562–0.9019)	0.4387 (0.3703–0.5045)	Not applicable
ICU/Fusion	0.9982 (0.9972–0.9990)	0.9925 (0.9884–0.9957)	0.0440
ICU/Vital	0.9961 (0.9942–0.9975)	0.9847 (0.9772–0.9901)	0.0467
ICU/ECG	0.9420 (0.9306–0.9534)	0.8003 (0.7589–0.8370)	0.0999
ICU/NEWS2	0.8131 (0.7916–0.8345)	0.4701 (0.4271–0.5232)	Not applicable

All 2000 stored-score encounters, including 48 quality rejections. AP is non-interpolated average precision. Model rows retain the original 800-resample intervals; post hoc NEWS2 intervals use 800 encounter resamples with seed 20260923. NEWS2 is an ordinal score, not a probability; a raw Brier score is therefore not assigned. Paired differences are in Supplementary Table S9.

**Table 3:** Prognostic sensitivity and specificity at the fixed thresholds.

Outcome/Input	Threshold	Sensitivity, % (95% CI)	Specificity, % (95% CI)	TP/FP/FN/TN
Mortality/Fusion	0.10	100.0 (98.1–100.0)	72.3 (70.2–74.4)	189/501/0/1310
Mortality/Vital	0.10	100.0 (98.1–100.0)	67.1 (64.9–69.3)	189/595/0/1216
Mortality/ECG	0.10	93.7 (89.2–96.7)	84.2 (82.4–85.9)	177/286/12/1525
ICU/Fusion	0.15	100.0 (99.0–100.0)	62.5 (60.1–64.9)	378/608/0/1014
ICU/Vital	0.15	100.0 (99.0–100.0)	60.5 (58.1–62.9)	378/640/0/982
ICU/ECG	0.15	97.6 (95.5–98.9)	65.6 (63.2–67.9)	369/558/9/1064

Two-sided exact 95% CIs. Positive if probability ≥ threshold. Recorded scores from rejected inputs are included; these results are not end-to-end usable-output sensitivity. TP, true positive; FP, false positive; FN, false negative; TN, true negative.

**Table 4:** Calibration and exploratory same-cohort recalibration.

Outcome/Input	Joint Intercept	Slope	CITL	O/E	Raw Brier	Apparent Brier
Mortality/Fusion	2.88	5.62	−0.602	0.684	0.0212	0.0043
Mortality/Vital	1.21	6.16	−0.818	0.609	0.0224	0.0047
Mortality/ECG	4.97	3.60	0.123	1.108	0.0594	0.0369
ICU/Fusion	1.00	6.09	−0.558	0.740	0.0440	0.0132
ICU/Vital	−0.15	4.47	−0.674	0.704	0.0467	0.0188
ICU/ECG	2.97	3.42	0.065	1.048	0.0999	0.0703

CITL, calibration-in-the-large from a separate offset model; O/E, observed/expected event ratio. Apparent Brier scores follow unpenalized logistic recalibration fitted and evaluated on the same encounters. They are not independently validated performance.

**Table 5:** Exploratory diagnostic performance at unchanged thresholds.

Measure	STEMI	ACS-Any
Events/*N*	11/2000	26/2000
Threshold	0.40	0.35
Sensitivity, % (95% CI)	90.9 (58.7–99.8)	92.3 (74.9–99.1)
Specificity, % (95% CI)	100.0 (99.8–100.0)	100.0 (99.8–100.0)
PPV, % (95% CI)	100.0 (69.2–100.0)	100.0 (85.8–100.0)
NPV, % (95% CI)	99.95 (99.72–100.00)	99.90 (99.63–99.99)
Miss rate, % (95% CI)	9.1 (0.2–41.3)	7.7 (0.9–25.1)
AUROC/AP	1.0000/1.0000	1.0000/1.0000
TP/FP/FN/TN	10/0/1/1989	24/0/2/1974

All 2000 encounters. Exact 95% CIs; narrow specificity intervals reflect the large non-event denominator, while sensitivity remains imprecise. Perfect sample ranking does not imply zero future diagnostic error. TP, true positive; FP, false positive; FN, false negative; TN, true negative.

## Data Availability

Participant-level clinical data and source ECG or monitor images are not publicly available because of privacy obligations and institutional access restrictions. Aggregate results are provided in the manuscript and Supplementary Materials. Access to participant-level data requires the applicable institutional and ethics approvals, an appropriate data-use agreement, and secure handling. Public release of analysis code, model source code, and weights has not been authorized.

## Supplementary Materials

The following supporting information can be downloaded at: https://www.mdpi.com/article/10.3390/diagnostics16193153/s1, Table S1: Complete operating-point estimates of recorded prognostic scores at the fixed thresholds; Table S2: Calibration of recorded prognostic probabilities by equal-rank risk decile; Table S3: Performance in accepted-input and quality-rejected encounters; Table S4: Triage classification performance before and after NEWS2-based fallback; Table S5: Field-specific OCR agreement and error; Table S6: Recorded runtime and memory summaries; Table S7: Outcome-stratified bootstrap confidence intervals (sensitivity analysis); Table S8: NEWS2 discrimination and threshold performance (NEWS2 ≥ 5 and ≥7) with exact confidence intervals and confusion counts; Table S9: Paired AUROC differences in all and accepted-input encounters; Table S10: Recoverability inventory of model-development methods; Table S11: Reporting inventory for cohort construction and eligibility; Table S12: Reporting inventory for image acquisition and failure handling; Note S1: Source-reported component benchmarks, including ECG digitization. Participant-level data and source ECG or monitor images are not included.

## Abbreviations

ACS, acute coronary syndrome; ACVPU, alert, new confusion, voice, pain, unresponsive; AI, artificial intelligence; AP, average precision; AUPRC, area under the precision–recall curve; AUROC, area under the receiver-operating-characteristic curve; CI, confidence interval; CITL, calibration-in-the-large; ECG, electrocardiogram; ED, emergency department; ESI, Emergency Severity Index; ICU, intensive care unit; MAE, mean absolute error; NEWS2, National Early Warning Score 2; NPV, negative predictive value; O/E, observed-to-expected event ratio; OCR, optical character recognition; PPV, positive predictive value; ROC, receiver operating characteristic; STEMI, ST-elevation myocardial infarction.
