# Supplementary material for: On-Device ECG and Vital-Sign Prediction of 24-Hour Mortality and Unplanned ICU Admission in Emergency Department Patients: A Retrospective Evaluation of MEDI-AI
Source: Diagnostics (Basel). 2026 Sep 28;16(19):3153. doi: 10.3390/diagnostics16193153 (PMC13664406; doi:10.3390/diagnostics16193153)
Supplement: Supplementary file 1 [file diagnostics-16-03153-s001.zip › diagnostics-4589535-supplementary.pdf]

Supplementary Materials

On-Device ECG and Vital-Sign Prediction of 24-Hour Mortality and Unplanned ICU Admission in Emergency Department Patients: A Retrospective Evaluation of MEDI-AI

Supplementary numerical results were recomputed from the supplied encounter-level data using the methods described in the main manuscript.

Table S1. Full prognostic operating-point performance.

Outcome / input	PPV, % (95% CI)	NPV, % (95% CI)	Miss rate, % (95% CI)	TP / FP / FN / TN
Fusion / Mortality	27.4 (24.1–30.9)	100.0 (99.7–100.0)	0.0 (0.0–1.9)	189/501/0/1310
Vital / Mortality	24.1 (21.2–27.3)	100.0 (99.7–100.0)	0.0 (0.0–1.9)	189/595/0/1216
ECG / Mortality	38.2 (33.8–42.8)	99.2 (98.6–99.6)	6.3 (3.3–10.8)	177/286/12/1525
Fusion / ICU	38.3 (35.3–41.5)	100.0 (99.6–100.0)	0.0 (0.0–1.0)	378/608/0/1014
Vital / ICU	37.1 (34.2–40.2)	100.0 (99.6–100.0)	0.0 (0.0–1.0)	378/640/0/982
ECG / ICU	39.8 (36.6–43.0)	99.2 (98.4–99.6)	2.4 (1.1–4.5)	369/558/9/1064

Sensitivity, specificity, thresholds, AUROC, and AP are in main Tables 2–3. All-row denominator is 2,000, including 48 rejected inputs. TP, true positive; FP, false positive; FN, false negative; TN, true negative.

Table S2. Fusion calibration by equal-rank decile.

Outcome	Decile	Mean predicted	Events / N	Observed %, 95% CI	O/E
Mortality	1	0.0255	0/200	0.0 (0.0–1.8)	0.000
Mortality	2	0.0357	0/200	0.0 (0.0–1.8)	0.000
Mortality	3	0.0434	0/200	0.0 (0.0–1.8)	0.000
Mortality	4	0.0518	0/200	0.0 (0.0–1.8)	0.000
Mortality	5	0.0628	0/200	0.0 (0.0–1.8)	0.000
Mortality	6	0.0769	0/200	0.0 (0.0–1.8)	0.000
Mortality	7	0.0978	0/200	0.0 (0.0–1.8)	0.000
Mortality	8	0.1299	0/200	0.0 (0.0–1.8)	0.000
Mortality	9	0.2022	3/200	1.5 (0.3–4.3)	0.074
Mortality	10	0.6561	186/200	93.0 (88.5–96.1)	1.418
ICU	1	0.0544	0/200	0.0 (0.0–1.8)	0.000
ICU	2	0.0763	0/200	0.0 (0.0–1.8)	0.000
ICU	3	0.0922	0/200	0.0 (0.0–1.8)	0.000
ICU	4	0.1103	0/200	0.0 (0.0–1.8)	0.000
ICU	5	0.1329	0/200	0.0 (0.0–1.8)	0.000
ICU	6	0.1636	0/200	0.0 (0.0–1.8)	0.000
ICU	7	0.2112	0/200	0.0 (0.0–1.8)	0.000
ICU	8	0.3118	9/200	4.5 (2.1–8.4)	0.144
ICU	9	0.5854	169/200	84.5 (78.7–89.2)	1.443

Outcome	Decile	Mean predicted	Events / N	Observed %, 95% CI	O/E
ICU	10	0.8152	200/200	100.0 (98.2–100.0)	1.227

Deciles use stable score rank and exactly 200 encounters per group. Ties can cross adjacent groups. These are not quantile-cut groups with unequal denominators.

Table S3. Quality-partitioned stored-score performance.

Outcome / input	Input quality	Events / N	AUROC	Sensitivity %, CI	Specificity %, CI
Fusion / Mortality	accepted	186/1952	0.9994	100.0 (98.0–100.0)	72.3 (70.2–74.4)
Fusion / Mortality	rejected	3/48	1.0000	100.0 (29.2–100.0)	73.3 (58.1–85.4)
Vital / Mortality	accepted	186/1952	0.9995	100.0 (98.0–100.0)	67.4 (65.1–69.6)
Vital / Mortality	rejected	3/48	1.0000	100.0 (29.2–100.0)	57.8 (42.2–72.3)
ECG / Mortality	accepted	186/1952	0.9655	93.5 (89.0–96.6)	84.3 (82.5–86.0)
ECG / Mortality	rejected	3/48	0.9185	100.0 (29.2–100.0)	80.0 (65.4–90.4)
Fusion / ICU	accepted	368/1952	0.9981	100.0 (99.0–100.0)	62.5 (60.1–64.9)
Fusion / ICU	rejected	10/48	1.0000	100.0 (69.2–100.0)	63.2 (46.0–78.2)
Vital / ICU	accepted	368/1952	0.9959	100.0 (99.0–100.0)	60.4 (57.9–62.8)
Vital / ICU	rejected	10/48	1.0000	100.0 (69.2–100.0)	68.4 (51.3–82.5)
ECG / ICU	accepted	368/1952	0.9416	97.6 (95.4–98.9)	65.5 (63.1–67.9)
ECG / ICU	rejected	10/48	0.9658	100.0 (69.2–100.0)	68.4 (51.3–82.5)
STEMI	accepted	10/1952	1.0000	90.0 (55.5–99.7)	100.0 (99.8–100.0)
STEMI	rejected	1/48	1.0000	100.0 (2.5–100.0)	100.0 (92.5–100.0)
ACS	accepted	24/1952	1.0000	91.7 (73.0–99.0)	100.0 (99.8–100.0)
ACS	rejected	2/48	1.0000	100.0 (15.8–100.0)	100.0 (92.3–100.0)

Accepted N=1,952; rejected N=48. Rejected-subset estimates are descriptive, not clinical outputs; CIs are exact for proportions. No curve CIs are presented for the rejected subgroup.

Table S4. Triage confusion matrices before and after fallback.

Stage	Reference	Normal	Urgent	Critical	Class F1
raw	Normal	548	211	169	0.585
raw	Urgent	325	224	153	0.334
raw	Critical	72	206	92	0.235
final	Normal	544	215	169	0.586
final	Urgent	321	228	153	0.336
final	Critical	64	214	92	0.235

Rows are reference classes; columns are predictions. Fallback changed 16 Normal predictions to Urgent (8 reference-Critical, 4 Urgent, 4 Normal), without changing Critical recall.

Table S5. OCR agreement and field-specific absolute error.

Field / error unit	Exact match %, CI	MAE, all	MAE, mismatch only	Mismatches
Heart rate, bpm	95.55 (94.55–96.41)	0.119	2.674	89
Systolic BP, mmHg	95.70 (94.72–96.55)	0.142	3.302	86
Diastolic BP, mmHg	95.50 (94.50–96.37)	0.070	1.567	90
MAP, mmHg	91.30 (89.98–92.50)	0.093	1.068	174
Respiratory rate, /min	93.90 (92.76–94.91)	0.086	1.402	122
SpO_2, percentage points	97.45 (96.66–98.10)	0.035	1.392	51
Temperature, °C	95.35 (94.33–96.23)	0.008	0.168	93
Glasgow Coma Scale	98.50 (97.87–98.99)	0.015	1.000	30

All fields have denominator 2,000. MAE is in the indicated field units, not a cross-unit pooled error. Source-summary discrepancies are preserved in the author reconciliation sheet. BP, blood pressure; MAP, mean arterial pressure; SpO_2, peripheral oxygen saturation; MAE, mean absolute error.

Table S6. Recorded application runtime profile.

Measure	Median	90th percentile	95th percentile	Maximum
Latency, ms	1235.0	1521.1	1602.1	1942.0
Memory, MB	184.4	197.0	200.2	214.6

Quantiles use linear interpolation over all 2,000 encounter-level values. Thermal-state counts: Nominal 1890/2000, Fair 97/2000, Serious 13/2000. The all-encounter 95th-percentile latency is 1602.1 ms, the same estimate used in the main manuscript. Subgroup quantiles have different denominators and must not replace this value.

Table S7. Outcome-stratified bootstrap sensitivity analysis.

Outcome / input	AUROC	95% CI	AP	95% CI
Fusion / Mortality	0.9994	0.9989–0.9999	0.9953	0.9907–0.9986
Vital / Mortality	0.9995	0.9990–0.9998	0.9956	0.9917–0.9985
ECG / Mortality	0.9647	0.9532–0.9746	0.7860	0.7331–0.8384
Fusion / ICU	0.9982	0.9973–0.9989	0.9925	0.9889–0.9956
Vital / ICU	0.9961	0.9943–0.9975	0.9847	0.9782–0.9902
ECG / ICU	0.9420	0.9293–0.9518	0.8003	0.7602–0.8344

800 resamples within outcome classes. These intervals condition on observed class sizes; ordinary encounter bootstrap intervals are used in the main manuscript. No base model is refitted.

Supplementary Note S1. Source-reported component benchmarks

The source summary reports ECG lead accuracy of 99.5%, waveform correlation of 0.982, QRS timing error of 2.1 ms, ST-segment error of 0.04 mV, and digitizer failure of 0.40%. It also reports fully offline operation and no repeat failures. These values are retained as supplied summary-level observations; this package does not independently reconstruct them from paired ECG waveforms or network logs. They are not substituted for an end-to-end clinical evaluation.

The tables report estimates calculated using the recomputation methods documented in the main manuscript.

Table S8. Revision-added NEWS2 operating characteristics.

Outcome / population / cutoff	Sensitivity %, 95% CI	Specificity %, 95% CI	PPV %, 95% CI	NPV %, 95% CI	TP/FP/FN/TN
Mortality / all / ≥5	92.1 (87.2–95.5)	64.2 (61.9–66.4)	21.1 (18.4–24.1)	98.7 (97.9–99.3)	174/649/15/1162
Mortality / all / ≥7	87.8 (82.3–92.1)	74.2 (72.1–76.2)	26.2 (22.8–29.8)	98.3 (97.5–98.9)	166/467/23/1344
ICU / all / ≥5	81.2 (76.9–85.0)	68.2 (65.9–70.5)	37.3 (34.0–40.7)	94.0 (92.5–95.3)	307/516/71/1106
ICU / all / ≥7	71.2 (66.3–75.7)	77.6 (75.4–79.6)	42.5 (38.6–46.5)	92.0 (90.5–93.4)	269/364/109/1258
Mortality / accepted / ≥5	91.9 (87.0–95.4)	64.2 (61.9–66.5)	21.3 (18.5–24.3)	98.7 (97.9–99.3)	171/632/15/1134
Mortality / accepted / ≥7	87.6 (82.0–92.0)	74.3 (72.2–76.4)	26.5 (23.0–30.1)	98.3 (97.4–98.9)	163/453/23/1313
ICU / accepted / ≥5	80.7 (76.3–84.6)	68.1 (65.7–70.3)	37.0 (33.6–40.4)	93.8 (92.3–95.1)	297/506/71/1078
ICU / accepted / ≥7	70.4 (65.4–75.0)	77.5 (75.3–79.5)	42.0 (38.1–46.1)	91.8 (90.2–93.3)	259/357/109/1227

All: N=2,000; mortality events=189, ICU events=378. Accepted: N=1,952; mortality events=186, ICU events=368. Positive means supplied NEWS2 score ≥ cutoff. Intervals are two-sided exact Clopper–Pearson intervals; denominators differ by metric. The supplied score was used unchanged. These are exploratory stored-score comparisons, not end-to-end workflow sensitivity or validated action thresholds.

Table S9. Revision-added discrimination and paired AUROC comparisons.

A. Supplied NEWS2 discrimination.

Outcome / population	Events/N	AUROC (95% CI)	AP (95% CI)
Mortality / all	189/2000	0.8801 (0.8562–0.9019)	0.4387 (0.3703–0.5045)
ICU / all	378/2000	0.8131 (0.7916–0.8345)	0.4701 (0.4271–0.5232)
Mortality / accepted	186/1952	0.8811 (0.8569–0.9041)	0.4491 (0.3793–0.5203)
ICU / accepted	368/1952	0.8101 (0.7860–0.8346)	0.4644 (0.4160–0.5190)

B. Fusion AUROC minus comparator AUROC.

Outcome / population	Comparison	ΔAUROC (95% CI)
Mortality / all	Fusion - NEWS2	0.119332 (0.097721–0.143263)
Mortality / all	Fusion - Vital	-0.000039 (-0.000450–0.000366)
ICU / all	Fusion - NEWS2	0.185134 (0.164124–0.205972)
ICU / all	Fusion - Vital	0.002114 (0.000632–0.003920)
Mortality / accepted	Fusion - NEWS2	0.118385 (0.095584–0.142422)
Mortality / accepted	Fusion - Vital	-0.000038 (-0.000514–0.000333)
ICU / accepted	Fusion - NEWS2	0.188089 (0.163998–0.211939)
ICU / accepted	Fusion - Vital	0.002258 (0.000619–0.004011)

AP is non-interpolated average precision. Intervals use 800 ordinary paired encounter bootstrap resamples, seed 20260923 reset per population; identical indices are shared by every score and both endpoints. Percentiles use linear interpolation. All 800 replicates were valid for each endpoint; none was single-class. Predictions were fixed, no model was refitted, and no multiplicity adjustment was applied. These intervals do not account for within-person clustering or model-development uncertainty. The all-encounter comparisons include 48 input-quality rejections and do not establish clinical utility.

Table S10. Recoverable study information and remaining methodological limitations.

Item	Recovered information and boundary
Cohorts and analysis unit	Development: 3,000 encounters in 2024; evaluation: 2,000 in 2025, with author-confirmed cross-year patient separation. Exact sampling dates and within-year unique-person count or clustering key were unavailable.
Available analysis fields	Age, sex, recorded vital signs, GCS, NEWS2, stored component/fusion scores, reference outcomes, quality flags, OCR values, and runtime fields were available. This is not a verified complete model-predictor list.
Evaluated model specification	Release v1.0.4 is reported. Complete architecture, preprocessing and missing-input rules, hyperparameter selection, external pretraining provenance, and the fitted fusion rule could not be reconstructed.
Threshold provenance	Mortality ≥0.10; ICU ≥0.15; STEMI ≥0.40; ACS-any ≥0.35. Values were used unchanged. Prespecification is source-author-reported; a dated freeze log, selection criterion, and exact selection subset were unavailable.
Index time and information ordering	Outcome definitions specify 24 hours after measurement. Linked measurement, ECG/photo acquisition, processing, deterioration, treatment, and ICU-decision times were unavailable; the operational anchor and predictor precedence cannot be established.
Acquisition and device	The author confirmed photographs of ECG tracings on actual monitors. iPhone model, iOS build, lead configuration, and evaluated-release acquisition settings were unavailable.
Input-quality rejection	1,952 accepted and 48 rejected. No reliable cause codes, repeat-capture linkage, or display/suppression logs were available; OCR mismatch rates are not rejection-cause counts.
NEWS2 construction	A complete supplied score was available for all encounters. Oxygen-scale choice, oxygen treatment, new confusion/ACVPU, and a derivation formula were absent. Scores were not reconstructed or converted into probabilities.
Triage reference	ESI v4 panel reference and stored Normal/Urgent/Critical classes were reported. The five-level ESI-to-three-class mapping and disagreement/indeterminate handling were not recoverable.
Case-mix characterization	Available demographic and physiological summaries were retained. Presenting diagnoses, major diagnostic categories, and comorbidities were not available for additional baseline characterization.
Runtime	All-encounter P95 latency is 1602.1 ms using linear interpolation, consistent in the main manuscript and Table S6. Recorded processing latency is not total capture-to-usable-result time.

This inventory reports the available source records, not a newly executed study protocol. A research plan, uncompleted method draft, generic device capability, or representative interface was not substituted for evaluated-release documentation. The analysis did not establish absence of leakage, a unique-person count, or independently validated clinical performance.

Supplementary Table S11. Cohort construction and eligibility: reported information and unrecoverable details.

Item	Available information	Reporting boundary
Development/evaluation periods	3,000 development encounters from 2024; 2,000 evaluation encounters from 2025.	Exact within-year dates and the sampling log were unavailable; full-calendar-year coverage is not asserted.
Sampling frame and random sample	45,078 adult ED visits; random evaluation sample of 2,000. No additional pre-sampling eligibility filtering was reported after frame definition.	43,078 not sampled is an arithmetic complement, not an exclusion count or a reconstruction of the sampling process.
Age	Observed evaluation ages: 18–95 years; the source sampling frame was described as adult ED visits.	The original numerical age rule used to construct the adult frame was not recoverable. Observed minimum age is not substituted for eligibility.
Unique patients/repeated visits	Patient-level de-duplication between the 2024 and 2025 cohorts was applied.	Unique-patient count and within-2025 repeat-encounter linkage were unavailable; analysis and resampling remain encounter-level.
Transfers and readmissions	No separate transfer/readmission variables or handling log were available.	Their frequency and any original selection rule cannot be stated; no new exclusions were imposed.
Baseline ICU status	Excluded from the definition of unplanned ICU transfer within 24 hours.	This endpoint rule is not interpreted as a documented whole-cohort exclusion count.
Incomplete source ECG/vital records	All required delivered numerical fields were populated; no numerical imputation was used.	Completeness of delivered rows does not establish completeness of historical acquisitions or enumerate source records absent before extract construction.
Poor-quality inputs	48/2,000 encounters had a rejection flag; 1,952 were accepted.	Rejected encounters remain in the primary stored-score denominator, not the accepted-input analysis; rejection is not a usable-output success.
Other clinical/technical exclusions	No additional eligibility filtering before sampling was reported; all 2,000 delivered encounters were analysed.	An independently reconstructed exclusion log was unavailable. Missing counts are not assumed to be zero.

This inventory adds reporting detail, not new patient selection, model fitting, outcome adjudication, or clinical validation. Figure 1 shows the known denominators. Dates, person counts, and handling rules were not imputed.

Supplementary Table S12. Acquisition, timing, image quality, and failed-input handling.

Parameter	Available information	Information not recoverable / implication
ECG image source	Photographs of ECG tracings displayed on actual monitors.	Original images and a complete acquisition log were not available for this analysis; no image-level reconstruction audit was performed.
Smartphone / operating system	The implementation is described as iPhone-based; recorded component and latency fields were analysed.	The particular smartphone model and OS build were unavailable. These were not inferred from the interface or latency.
ECG lead configuration	The representative interface includes generic 12-lead guidance.	The configuration actually used for study acquisitions was unavailable; interface guidance is not acquisition evidence.
Measurement, capture and index time	The specified endpoint horizon was 24 hours after measurement.	Linked ECG/vital measurement, image-capture, processing, deterioration, treatment and ICU-decision times were unavailable; a verified prediction-time anchor cannot be reconstructed.
Images per encounter	The analysis contains 2,000 encounter-level records.	Image and attempt counts per encounter were unavailable. An encounter is not assumed to equal one image or one attempt.
Acquisition personnel	No operator-role or training log was available.	Nurse, physician, technician, investigator, or patient acquisition is not presumed.
Repeat image capture	No complete recapture policy or attempt-level history was available.	Permission, frequency, and success of repeat capture were not recoverable; stored rejected-input scores do not establish successful recapture.
Image-quality assessment	Only the final binary acceptance/rejection flag was available: 1,952 accepted and 48 rejected.	Assessment method, human/automated assignment, quality metrics, and cutoffs were unavailable. Cause-specific rejection counts cannot be reported.
Failed or rejected acquisition	All stored scores were retained for the labeled all-encounter benchmark; accepted-input analyses were separate.	No-output attempts, display/suppression, overrides, and actual fallback actions were not recoverable. Rejected scores are not treated as usable clinical outputs.
Vital signs and runtime	Recorded reference/OCR fields and latency were available for the reported component analyses.	These fields do not establish every monitor-acquisition step, predictor-availability time, or total capture-to-result time.

Missing information is reported as unavailable, not assumed absent. This table specifies the limits of the retrospective extract and must not be interpreted as a reconstructed prospective acquisition protocol.
